# Fat necrosis: A consultant’s conundrum

**DOI:** 10.3389/fonc.2022.926396

**Published:** 2023-02-16

**Authors:** Jinita Majithia, Purvi Haria, Palak Popat, Aparna Katdare, Sonal Chouhan, Kunal Bharat Gala, Suyash Kulkarni, Meenakshi Thakur

**Affiliations:** Radiology Department, Tata Memorial Hospital, Mumbai, India

**Keywords:** fat necrosis, breast imaging, radiology, mammography, ultrasonography

## Abstract

Fat necrosis of the breast is a benign non-suppurative inflammation of the adipose tissue and often mimics breast cancers, posing a diagnostic challenge for the clinician and radiologist. It has a myriad of appearances on different imaging techniques, ranging from the pathognomic oil cyst and benign dystrophic calcifications to indeterminate focal asymmetries, architectural distortions, and masses. A combination of different modalities can assist a radiologist in reaching a logical conclusion to avoid unnecessary interventions. The aim of this review article was to provide a comprehensive literature on the various imaging appearances of fat necrosis in the breast. Although a purely benign entity, the imaging appearances on mammography, contrast-enhanced mammography, ultrasound, and magnetic resonance imaging can be quite misleading, especially in post-therapy breasts. The purpose is to provide a comprehensive and all-inclusive review on fat necrosis with a proposed algorithm allowing a systematic approach to diagnosis.

## Introduction

Fat necrosis of the breast is a benign non-suppurative inflammation of the adipose tissue (1). It often mimics breast cancer and poses a diagnostic challenge for the clinician and radiologist. In the majority of cases, imaging provides conclusive evidence of its benignity; however, in a small percentage of cases, histological sampling becomes necessary to exclude malignancy, owing to its close semblance on imaging.

The breast parenchyma, which is composed of adipose, epithelial, and stromal tissues, is enveloped by skin and subcutaneous tissue. Adipose tissue forms the majority of the bulk of the breast volume, and the amount of adipose tissue varies throughout the reproductive life of a woman. An injury to the adipose tissue results in fat necrosis.

This review article aims to discuss and illustrate the spectrum of appearances of fat necrosis using different imaging techniques. In this article, we also review the literature on the clinical features and etiopathogenesis relevant to a radiologist. The purpose is to provide a comprehensive and all-inclusive review on fat necrosis with a proposed algorithm allowing a systematic approach to diagnosis.

## Pathophysiology

The most common stimulus for fat necrosis is hypoxia, leading to ischemia. The fragmentation of adipose cells following ischemic damage leads to the formation of intracellular vacuoles filled with necrotic lipid material. Fibroblasts, multinucleated giant cells, and lipid-laden or foamy histiocytes (“fat-filled macrophages” or “foam cells”) along with enucleated adipocytes begin to accumulate (2). Damage to adipose cells also releases lipase in the interstitium which leads to triglyceride breakdown and the release of fatty acids. When unresolved, this leads to a cavity formation due to liquefactive necrosis, also known as membranous fat necrosis. Simultaneous fibrinogen secretion in the interstitium from the damaged blood vessels is followed by conversion to active fibrin catalyzed by thrombin (3), which is key to the development of fibrosis. Thus, loculated necrotic fat within the cystic cavity eventually gets surrounded by dense fibrous tissue. Sometimes, the negatively charged fatty acids bind to the positively charged calcium ions in a process called saponification (4), which leads to the development of calcification within the fat necrosis.

Irreversible cell injury is of two types: apoptosis and necrosis. In the breast, both these processes prevail. The intensity of initial insult determines which process will predominate which in turn determines the clinical presentation, the radiological appearance, and the histological finding (2). The greater the necrotic component, the greater the inflammation and the worse the clinical condition (3). Thus, a close correlation exists between the clinical age of the lesion, radiological appearances, and expected gross and histological findings in breast fat necrosis. All of these vary based on the time lapse from an inciting event.

## Etiology

The common etiological factors leading to breast fat necrosis include trauma (accidental or iatrogenic), radiotherapy, systemic anticoagulation therapy like warfarin, infection, and idiopathic disease. The most common cause is accidental trauma accounting for 21%–70% of all cases of fat necrosis (2). Seat-belt injury is the most common type of blunt accidental trauma. Iatrogenic causes of trauma include interventions like cyst aspirations, incisional or excisional biopsies, and vacuum-assisted biopsies (VAB). Idiopathic fat necrosis of the breast is common in fatty pendulous breasts of middle-aged women (5).

Various surgeries, including but not limited to lumpectomy, breast conservative surgery (BCS), mastectomy, reduction mammoplasty, implant removal, and breast reconstruction, increase the risk of fat necrosis in the breast. A recent study by Dolan et al., comparing the imaging and biopsy results after breast surgery, found that the rate of fat necrosis after oncoplastic BCS was 7% as confirmed by pathology (6). Fat necrosis in the flap following reconstruction surgeries occurs due to ischemia from inadequate arterial inflow or poor venous outflow and is dependent on the type of flap (pedicled tissue flap *vs*. free fat flap), the surgeon’s experience, and the administration of adjuvant radiation. Nakada et al. reported a 39% incidence of fat necrosis following pedicled tissue flap and almost 100% following free dermal fat flaps. Furthermore, symptomatic fat necrosis also showed a lower incidence following pedicled tissue flaps (2.9%) as compared with the free flaps (25%) (7). A confounding factor associated with fat necrosis following free flaps was smoking, and often the surgeons required patients to quit smoking at least 8 weeks prior to surgery (8–10). Fat grafting, which is used as a cosmetic procedure following BCS, involves harvesting fat from one part of the body and injecting it at the site that needs correction of the contour deformity. This leads to random diffusion and neovascularization of the grafted fat globules leading to fat necrosis. The incidence of fat necrosis following fat grafting varies from 2% to 18% (11).

Alone or following BCS, radiotherapy is an independent risk factor for the development of fat necrosis secondary to inflammation (12). The observation that recurrences tend to occur commonly at or near the previous lumpectomy site has led to the widespread use of accelerated partial breast irradiation (APBI) which delivers a larger dose per fraction over a shorter period of time to a targeted portion of the breast, i.e., the tumor bed, instead of the entire breast just like in whole-breast radiation (13). Brachytherapy, which can be used by itself as a form of APBI, is of two types: intracavitary and interstitial. Interstitial brachytherapy is delivered using hollow needles implanted in the tumor bed with radioactive pellets inserted through them at the time of radiotherapy (14). There is some evidence that interstitial brachytherapy causes additional trauma to the breast parenchyma from the implanted needles and, thus, leads to a higher incidence of fat necrosis. A similar incidence of symptomatic and asymptomatic fat necrosis has been reported following conventional WBI and APBI brachytherapy; however, it is greatly influenced by the volume of the irradiated breast as well as the strength and duration of irradiation (15). Different studies have reported a variable crude incidence of fat necrosis following radiation therapy in early-stage breast cancer. Wazer et al. found that the crude incidence for clinically evident fat necrosis was 27% (16). Garsa et al. studied 238 breasts in 236 women and reported that the crude incidence of fat necrosis was 17.6% and the rate of symptomatic fat necrosis was 10.1% (13). The median time to the development of fat necrosis following radiation to the breast was found to be 12.7 months (average range 3–42 months) by Rahimi et al. (17).

## Clinical features

Fat necrosis is more often than not asymptomatic and diagnosed incidentally on imaging. The clinical findings do not normally vary according to the etiology of fat necrosis and are neither specific nor sensitive. Palpable lumps of fat necrosis may present as indolent nodular and mobile masses with smooth margins or as hard fixed irregular masses. Associated features like induration, ecchymosis, erythema, nipple retraction, skin retraction or dimpling, and lymphadenopathy may be present. More than 50% of symptomatic fat necrosis has clinical features of malignancy such as hard mass, nipple retraction, and skin tethering (18). Lesions developing following trauma are usually at or near the site of trauma, and when no relevant history is found, the lesions were most commonly located in the upper outer quadrant (18). Fat necrosis in obese women with pendulous breasts was commonly seen in the superficial and subareolar tissues (5).

## Imaging

The imaging modalities for the diagnosis of fat necrosis in the breast include mammography (MMG), ultrasound (USG), and magnetic resonance imaging (MRI). The emerging newer technique of contrast-enhanced mammography (CEM) aids in the diagnosis of fat necrosis in disputed cases and, along with MRI, serves as a road map for targeted biopsies.

### Mammography

Mammography, digital mammography (DM), or digital breast tomosynthesis (DBT) plays a pivotal role in the imaging of fat necrosis especially in clinically suspicious symptomatic women above the age of 40 years and in post-therapy cases. In fact, in a postoperative and post-therapy breast, a regular annual follow-up mammogram is the gold standard for imaging surveillance (19). It should be borne in mind, however, that a normal mammogram does not rule out an underlying pathology especially in dense breasts due to the overlap of lesions by glandular parenchyma. In such cases, USG is performed as a complementary investigation tool.

The appearance of fat necrosis on mammogram is in concordance with the stage of evolution of fat necrosis and ranges from focal asymmetries, architectural distortions, mass-forming solid lesions, cystic lesions, oil cysts, and calcifications. Early lesions develop hemorrhagic foci or areas of indurated fat with trabecular edema and can appear as focal asymmetry on MMG (12). Occasionally, like in post-traumatic cases, a hematoma formation is seen which evolves into a seroma, both appearing as small isodense mass-forming lesions on MMG. Some lesions develop a central cavity with liquified and necrotic contents called membranous fat necrosis also appearing as isodense lesions on MMG.

A loculated necrotic fat-containing cavity called an oil cyst appears as a radiolucent lesion on MMG, owing to the internal fat component, with a thin dense peripheral rim of fibrosis. Oil cysts are pathognomic of fat necrosis and are the second most prevalent finding on MMG after dystrophic calcifications, accounting for 27% of the cases (20). The fibrous rim of an oil cyst may calcify over time forming a thin dense rim of calcification, formerly called as “egg-shell calcification.” The rim calcification does not develop entirely at the same time with inception as small foci of calcification, leading to curvilinear or arc-like calcification and eventually progressing into a complete rim. Thus, in the early stages, the small foci of calcification in the wall of an oil cyst appear similar to fine microcalcifications and need differentiation from the disease process (**Supplementary Figures 1, 2**). In certain instances, when the oil cysts are not purely fat-containing and have fat–fluid or fat–blood levels within, ultrasound serves as a problem-solving tool. With the incomplete replacement of fat and associated intense fibrotic reaction surrounding the oil cyst, thickened irregular walls may develop around the residual necrotic fat, giving a spiculated appearance on MMG, mimicking cancer (12). MMG is usually sufficient for the diagnosis of oil cysts, warranting no further investigation or follow-up; however, it is important to note that oil cysts may occasionally be occult on MMG, especially when overlapped by normal fatty and fibroglandular breast parenchyma and get diagnosed on USG or MRI. **Supplementary Table 1** summarizes the key points of oil cysts.

An intermediate to late presentation of fat necrosis on MMG is focal asymmetry or architectural distortion. DBT is most useful in such cases to decrease the confounding effect of overlapping breast tissue. The underlying pathophysiology is the presence of varying amounts of inflammatory changes and areas of fibrosis interspersed with radiolucent necrotic fat. These are usually not clinically palpable and, in the majority of cases, are diagnosed solely on imaging. A post-surgical scar may appear as an area of architectural distortion with overlying contour deformity and skin thickening or nipple retraction at the site of surgery, features that may also mimic recurrence (**Supplementary Figure 3**). The identification of interspersed fat within an asymmetry can increase the confidence levels for benignity; however, existing fat within the breast can be engulfed by an evolving malignant process and must be viewed with suspicion and evaluated further, either with DBT, CEM, or MRI, and further histological confirmation as required (21).

Calcification is one of the most common findings following lumpectomy and radiation and also the most important imaging biomarker for local recurrence (22). With a rising trend toward breast conservation, there has been a learning curve with the imaging evaluation of post-therapy calcifications, to be able to adequately differentiate benign calcifications of fat necrosis from malignant microcalcifications of residual or recurrent disease (23). It is observed that the median time for the development of benign calcifications is much earlier than malignant calcifications. Günhan-Bilgen et al. (24), Giess et al. (23), and Chang Sen et al. (25) reported that the median times for the development of benign calcifications were 24, 23, and 27 months, respectively, and for malignant calcifications, the median times were 52, 39, and 41 months, respectively. Therefore, a lower probability of malignancy is observed with early developing calcification (6–24 months) (23). The incidence of benign calcifications was also observed to be higher than malignant microcalcifications in post-therapy breasts (23, 24, 26). In the majority of cases, the calcifications of fat necrosis occur in and around the area of surgery, usually within the same quadrant (22, 27). Thus, calcifications observed elsewhere in the breast or in the contralateral breast should be addressed with caution (**Supplementary Figure 4**).

Calcifications representing recurrence commonly have an amorphous or fine pleomorphic morphology with segmental or regional distribution (28). Calcifications of early-stage fat necrosis may also appear fine and pleomorphic closely mimicking cancer; however, they show gradual coarsening with evolution into dystrophic calcifications (**Supplementary Figure 5**). Although not a ground rule, fine microcalcifications may be differentiated by the presence of fat-density radiolucent areas around and within the calcifications in fat necrosis, whereas the presence of high density associated with calcification is suggestive of recurrent disease (29). Unless unequivocally benign, all post-therapy calcifications should be viewed with caution. It is imperative to emphasize that suspicious-looking calcifications must undergo tissue diagnosis (**Supplementary Figure 6**).

The most common calcification in fat necrosis is dystrophic calcifications. These are larger than 1 mm, rough, and irregular and tend to coalesce to become larger. Calcifications within the irradiated breast are usually dystrophic with a typical benign appearance. They appear linear or round and coarse, within the irradiated field. The tubular appearance of these calcifications following brachytherapy is secondary to the fat necrosis developing along the implanted needle tract which on serial MMG shows classical interval coarsening (**Supplementary Figure 7**). The calcifications that develop in silicone granulomas or after autologous fat grafting are also coarse and dystrophic (**Supplementary Figure 8**). As mentioned, rim calcification in the wall of the oil cyst is also typically benign. The key points of calcifications in a post-therapy breast are summarized in **Supplementary Table 2**.

### Ultrasonography

Ultrasound is a well-established, quick, and effective modality for imaging the breast for fat necrosis. The absence of hazardous radiation makes it the preferred investigation tool for symptomatic women less than 30 years of age as well as pregnant and lactating women. The sensitivity of USG is higher than mammograms especially in dense breasts of women less than 50 years of age (30, 31); however, MMG and USG are most effective when used in combination with the highest diagnostic accuracy when utilized together (31). Complemented with color Doppler and elastography, ultrasound is meritorious in differentiating benign fat necrosis from malignant lesions by allowing non-invasive characterization of tissue vascularity and stiffness, respectively. The absence of color flow on color Doppler hints toward the benignity of fat necrosis, but it is not reliable (32). On elastography, malignant lesions are expected to be hard, and benign lesions are presumed to be soft; however, a classic example of a confounding finding on elastography is the increased stiffness of benign lesions such as fibrosis and fat necrosis (33).

Fat necrosis on ultrasound may appear as solid or cystic masses. The solid masses of fat necrosis have well-circumscribed margins and may distort breast parenchyma. The cystic lesions may have clear contents, internal echoes, and fluid–debris levels or may appear as complex intracystic masses. The common appearances of oil cyst on USG include anechoic cystic lesions with posterior acoustic enhancement or anechoic lesions with posterior acoustic shadowing (34). The typical oil cysts on mammograms often appear as solid masses on USG (34) (**Supplementary Figures 9, 10**). An internal echogenic band, formed by the interface between lipid and serous/hemorrhagic fluid, that shifts its orientation with a change in patient position is a hallmark of oil cysts (34). With increasing complexity, internal echogenic mural nodules, thick septations, or calcifications may be seen (35). Conventionally, malignant hypoechoic masses are expected to demonstrate posterior acoustic shadowing; however, oil cysts, dystrophic calcifications, and focal architectural distortions of fat necrosis may also show dense posterior shadowing (36).

Increased echogenicity of surrounding breast parenchyma or subcutaneous fat is a reliable indicator of benignity (32). Fat necrosis, especially when precipitated from trauma, is superficial in location and appears as a hyperechoic mass with or without a small central hypoechoic focus (**Supplementary Figures 11, 12**). Only a small proportion, less than 0.8%, of hyperechoic masses represent malignancy (37). When situated deeper in the fibroglandular parenchyma of the breast, a hyperechoic mass needs to be viewed with caution and warrants differentiation from malignant lesions, such as lymphoma, leukemia, metastasis, intralobular carcinoma, or rarely intraductal carcinoma (37).

### Contrast-enhanced mammography

CEM is a novel imaging modality developed as an adjunct to mammography to provide additional physiologic information about local breast perfusion. An extensive search of the medical database revealed no publications describing or reviewing the appearance of fat necrosis on CEM. Very little literature was found on the benefits of CEM in the evaluation of mass-forming lesions, calcifications, or architectural distortions.

One of the common indications of CEM is a palpable mass in a postoperative breast. Mixed-density lesions on mammogram and ultrasound may need further characterization with CEM (**Supplementary Figure 10**). A heterogeneous area of intermixed fibroglandular and fatty tissue either shows no enhancement or shows thin uniform peripheral and/or septal enhancement (38). An oil cyst with a fibrous rim also shows thin uniform peripheral enhancement on CEM. For the assessment of calcifications, CEM may be beneficial in differentiating benign calcification of fat necrosis from suspicious microcalcifications by the absence of enhancement. The presence of enhancement supports the diagnosis of malignancy; however, the absence does not exclude it (39, 40). The interpretation of architectural distortion secondary to fat necrosis can be rather challenging on imaging. Although distortions are better evaluated on DBT, it is questionable whether it can obviate the need for biopsy owing to the low positive predictive value of DBT, especially when no ultrasound correlate is found (41). CEM can be of particular value in such cases as architectural distortion or focal asymmetry from fat necrosis usually does not demonstrate enhancement on CEM. A few benign causes of architectural distortions like a radial scar or complex sclerosing lesions, sclerosing adenosis, and post-surgical changes are close differentials. CEM may not always prove to be beneficial in equivocal cases for differentiating benign and malignant architectural distortions, and the paucity of data often compels the radiologist to consider biopsy in many cases of architectural distortion irrespective of demonstrable enhancement on CEM (42).

### Magnetic resonance imaging

MRI has been a game changer in breast imaging owing to its high soft tissue resolution. It is not routinely required while evaluating breasts for fat necrosis; however, in some post-therapy complicated cases, when mammography and ultrasound findings are ambiguous and the clinical suspicion is high, the utility of MRI is justified.

The T1-weighted sequence (T1W) is one of the most important sequences for the diagnosis of fat necrosis. Demonstration of fat-containing lesions and tissue distortion in the operative bed (fat-engulfing scar tissue) on T1W sequence can significantly improve the diagnostic conviction even in the presence of suspicious enhancement or kinetics which can be misleading (43). Fat-saturated T1W images can confirm the fatty nature of the lesion. Diffusion-weighted imaging (DWI) with apparent coefficient of diffusion (ADC) map provides quantitative and qualitative data for differentiating postoperative fat necrosis from recurrence and improves the overall diagnostic accuracy of MRI breast (43, 44). Visualization of bright signal intensity in the postoperative bed with increasing *b*-values and low-signal intensity on the ADC map corresponds to recurrent disease (44). The kinetic curve assessment following dynamic post-contrast image acquisition may help in distinguishing benign from malignant causes of enhancement; however, it is non-specific and varies from slow and gradual to rapid enhancement (44).

Post-treatment or post-traumatic breasts with early-onset hemorrhage at the local site show a well-defined or an ill-defined mass or a focal asymmetry with altered signal intensity on different sequences. The signal intensity varies based on the age of blood, typically hyperintense on T1W images and hypointense on T2W images. No enhancement or mild peripheral enhancement can be seen on the post-contrast sequence (45). One of the most common MRI findings of fat necrosis is of an oil cyst, with a well-defined round to oval lesion with T1W and T2W hyperintense contents, following fat signal intensity on all sequences. Suppression of the signal on fat-saturated (FAT-SAT) T1 and short tau inversion recovery (STIR) images confirms the presence of fat within the lesion. The thin fibrous rim of an oil cyst shows subtle uniform enhancement (**Supplementary Figures 13, 14**). A signal drop in the rim of the oil cyst is suggestive of rim calcification. Fat necrosis showing decreased signal intensity on T1- and T2-weighted images can be a result of iron-containing siderophages (46, 47).

Architectural distortions occurring at or near the lumpectomy site under ideal circumstances would show signal intensity similar to fat on all sequences with adjacent parenchymal enhancement (48, 49). It can be quite a challenge to interpret architectural distortions, especially in the early postoperative phases because of the intense enhancement seen due to acute inflammation and edema. Mild mass-like enhancement is usually seen lasting for up to 18 months in the postoperative and post-radiation breast (50). A minimal or a small focal area of enhancement or thin linear homogeneous non-mass enhancement (NME) can be seen persisting for up to 5 years post-lumpectomy (51). Fibrosis, which is often identified in conjunction with fat necrosis, leads to the development of an irregular mass or architectural distortion and focal asymmetry with varying appearances on the T1W sequence (52). Multiple enhancement patterns of fibrosis are identified on MRI correlating with the stage of evolution of fat necrosis in a post-therapy breast. More recent lesions generally have an irregular contour with variable enhancement surrounding the lesion, whereas older lesions have markedly irregular margins, owing to fibrosis and retraction, and generally do not enhance (53) (**Supplementary Figure 14**). Fibrosis usually shows persistent or delayed plateau kinetics.

Certain patterns of enhancement are highly suspicious, like mass-like enhancement, nodular enhancement (more than 5 mm), and clumped or heterogeneous non-mass enhancement with segmental or regional distribution and suspicious kinetics (like rapid initial enhancement and washout), and should not be considered as benign fat necrosis.

### Positron emission tomography/computed tomography

The imaging features of fat necrosis on positron emission tomography/computed tomography (PET/CT) are all incidentally detected. ^18^F-FDG PET/CT is not a routine recommendation for the detection of breast abnormalities and is primarily done for staging and metastatic evaluation. It cannot be emphasized enough that PET/CT is neither indicated nor recommended for recurrent disease evaluation in post-therapy breasts.

Fat necrosis shows no uptake on PET/CT; however, few studies have detected “false positive” cases demonstrating uptake on PET/CT (47, 54). The increased FDG uptake of fat necrosis can be attributed to the presence of locally increased metabolically active inflammatory cells reflecting hyperemia (2) (**Supplementary Figure 15**). Intense uptake in the setting of transverse rectus abdominis myocutaneous (TRAM) flap reconstruction is seen when the fat-rich tissue is damaged intraoperatively (55). Other benign conditions showing FDG uptake that may sometimes need differentiation from fat necrosis are acute and chronic inflammation.

## Tissue diagnosis

Cases that are clinically and radiologically equivocal require confirmation with tissue diagnosis. Minimally invasive methods for tissue sampling are fine needle aspiration cytology (FNAC), core needle biopsy, and vacuum-assisted biopsy (VAB). The sensitivity and specificity of FNAC are 87% and 99%, respectively; however, it bears limitations such as inadequate sampling and repeated needling (2). Core needle biopsy has a higher sensitivity and diagnostic accuracy, almost comparable to surgical biopsy, and also allows immunohistochemistry testing of the tissue. The false-negative rate of core biopsy is 1.2% to 1.5% (56); thus, in a small proportion of cases when clinical suspicion is high, a surgical biopsy is recommended despite a negative core biopsy.

A spiculated dense mass on mammogram and a hypoechoic mass with angular margins and a taller-than-wide appearance on ultrasound in a post-therapy breast are suspicious features and warrant a tissue diagnosis. Amorphous or fine pleomorphic microcalcifications also require a histological sampling, compared with dystrophic and rim calcifications which are classically benign.

A summary of the different stages of fat necrosis is tabulated with its clinical and radiological features as well as its gross histopathological and microscopic features in **Table 1**.

**Table 1:** Clinical-Radiological-Pathological correlation with underlying pathology in Fat Necrosis.

Clinical	Stage	Radiological	Gross Pathological	Microscopic
Asymptomatic*I* Induration / Firmness/ Palpable Lump (tenderness+/-)	Early	Focal asymmetry or isodense mass-forming lesion	Haemorrhagic foci in the breast or areas of indurated fat. Bright yellow fat (Saponification)	Haemorrhage within fat with enucleated adipocytes,foamy histiocytes and multinucleated giantcells (due to phagocytosisof necrotic adioocvtes)
	Intermediate to Late	Isodenselesion corresponding to a cystic lesion	Cystic lesion filled with liquefied content	Cavity formation due to liquefactive necrosis, known as membranousfat necrosis
		Oil Cyst: Fat density lesion with well-defined margins Peripheral rim of calcification+/-	Cavitary lesion with firm/gritty walls and soft necrotic contents "membranousfat necrosis"	Loculated necrotic fat within a cyst surrounded by dense fibrous tissue. Egg-shell calcification +/-
		Micro or macrocalcification	Chalky white gritty areas (Calcification)	Specks or massesof dystrophic calcification± giant cell reaction
		!Asymmetry *I* Architectural distortion	Yellow-Grey firm areasof Fibrosis	Reactive inflammatory response,hemosiderin laden macrophages, areasof fibrosis and eventual scar formation

## Approach to the diagnosis of fat necrosis

Mammography has stood the test of time for imaging the breast in eligible women. It is the best and first investigation tool for most cases. The diagnostic accuracy of mammogram increases when combined with ultrasound (31). Ultrasound is the first investigation tool for women under 30 years of age (57). It is also the most common modality used for guided interventions. MRI is usually reserved for complicated cases when MMG and USG yield ambiguous results. However, MRI can be used as a frontline tool for imaging dense breasts, especially in young women less than 40 years of age (58).

**Figure 1:**
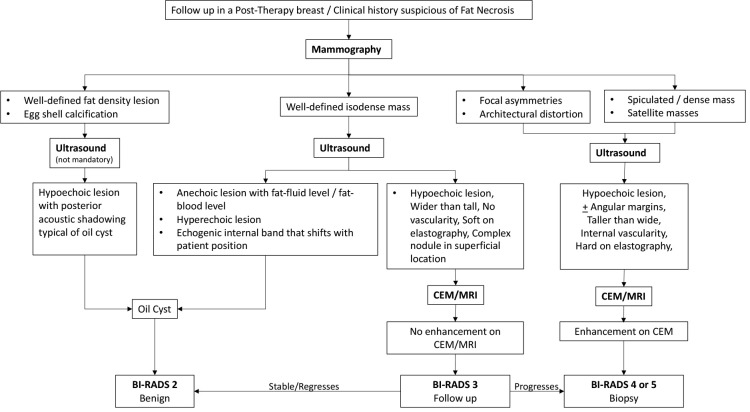
Algorithm for the approach to fat necrosis.

**Figure 2:**
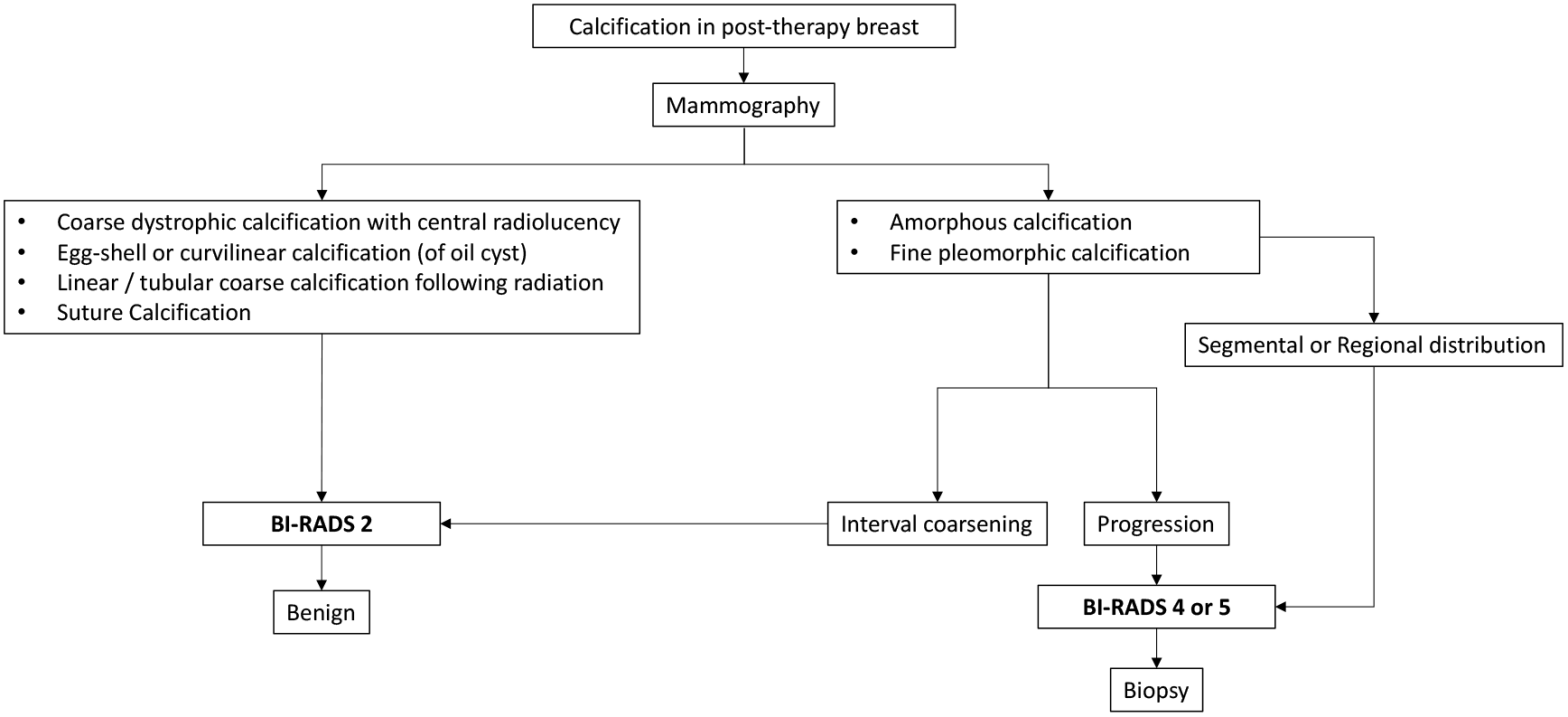
Algorithm for the approach to calcifications seen in fat necrosis.

The following flowcharts aspire to serve as a road map as an approach to various possible imaging appearances of fat necrosis in the post-therapy setting or a clinical setting of high suspicion for fat necrosis such as with a history of trauma, as depicted in **Figure 1**. The approach to calcifications in a post-therapy breast has also been depicted in a flowchart for ease of understanding in **Figure 2** and for interpretation of various morphologies and distribution of calcifications.

## Conclusion

Fat necrosis in the breast, albeit a benign entity, is a cause of concern for the clinician and often a cause of anxiety for the patient, and owing to its myriad of appearances on various imaging modalities, fat necrosis may be a cause of diagnostic dilemma for the radiologist. A radiologist should be conversant with the many typical and atypical features of fat necrosis and bear knowledge of the different evolution patterns enabling early diagnosis to circumvent unnecessary intervention. The overlap in imaging features of fat necrosis with breast cancer in a few cases makes it extremely difficult to reach a confident diagnosis based on imaging alone and often warrants histological sampling. Once diagnosed, fat necrosis requires no further attention or intervention as it bears no risk of malignant transformation.

With this comprehensive review article, one can attain information on the interplay of various imaging modalities such as MMG, USG, CEM, and MRI and their use in different permutations and combinations in a nutshell to aid in arriving at a logical conclusion for the diagnosis of fat necrosis. The one-of-a-kind tabulated information on the clinical–radiological–pathological correlation for different stages of fat necrosis makes it easy for a radiologist to better interpret the imaging findings based on the clinical presentation and expected cellular evolution. A visually stimulating flowchart for the approach to a post-therapy breast as well as calcifications allows one to become a clinical radiologist.

## Author contributions

JM is the lead author of this review and has written and given the maximum contribution to the manuscript. PH has been a guide and mentor and has reviewed the content in-depth. PP, AK, and SC have helped by providing the images for the representative cases and by providing their valuable inputs. KB and SK have helped with the image-guided interventions of the representative cases. MT is the corresponding author and a source of inspiration and guidance for writing the manuscript. All authors contributed to the article and approved the submitted version.
