# Supplementary material for: Fat necrosis: A consultant’s conundrum
Source: Front Oncol. 2023 Feb 16;12:926396. doi: 10.3389/fonc.2022.926396 (PMC9978799; doi:10.3389/fonc.2022.926396)
Supplement: Supplementary file 1 [file Presentation_1.pptx]

## Slide 1
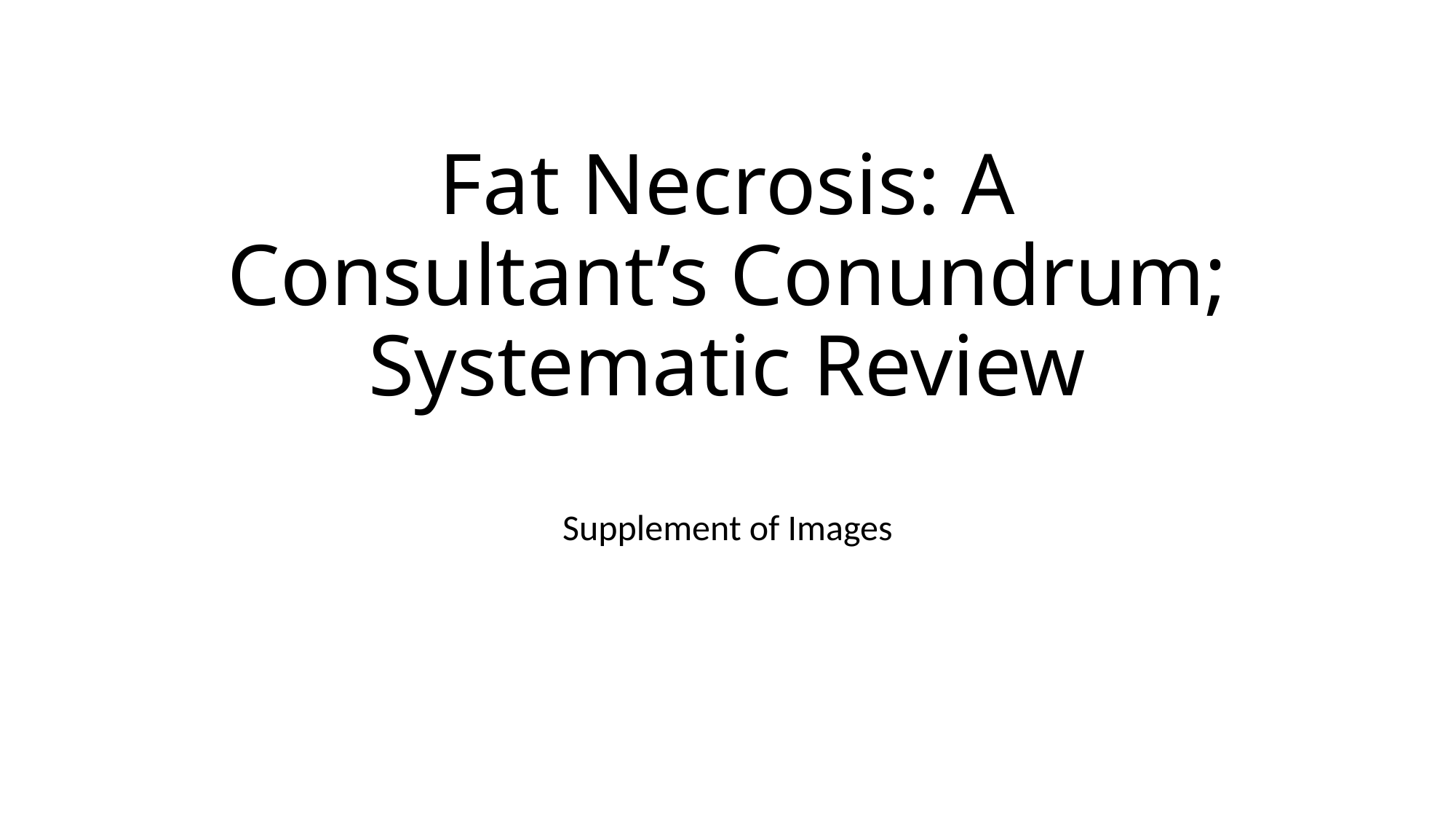

# Fat Necrosis: A Consultant’s Conundrum; Systematic Review
Supplement of Images

## Slide 2
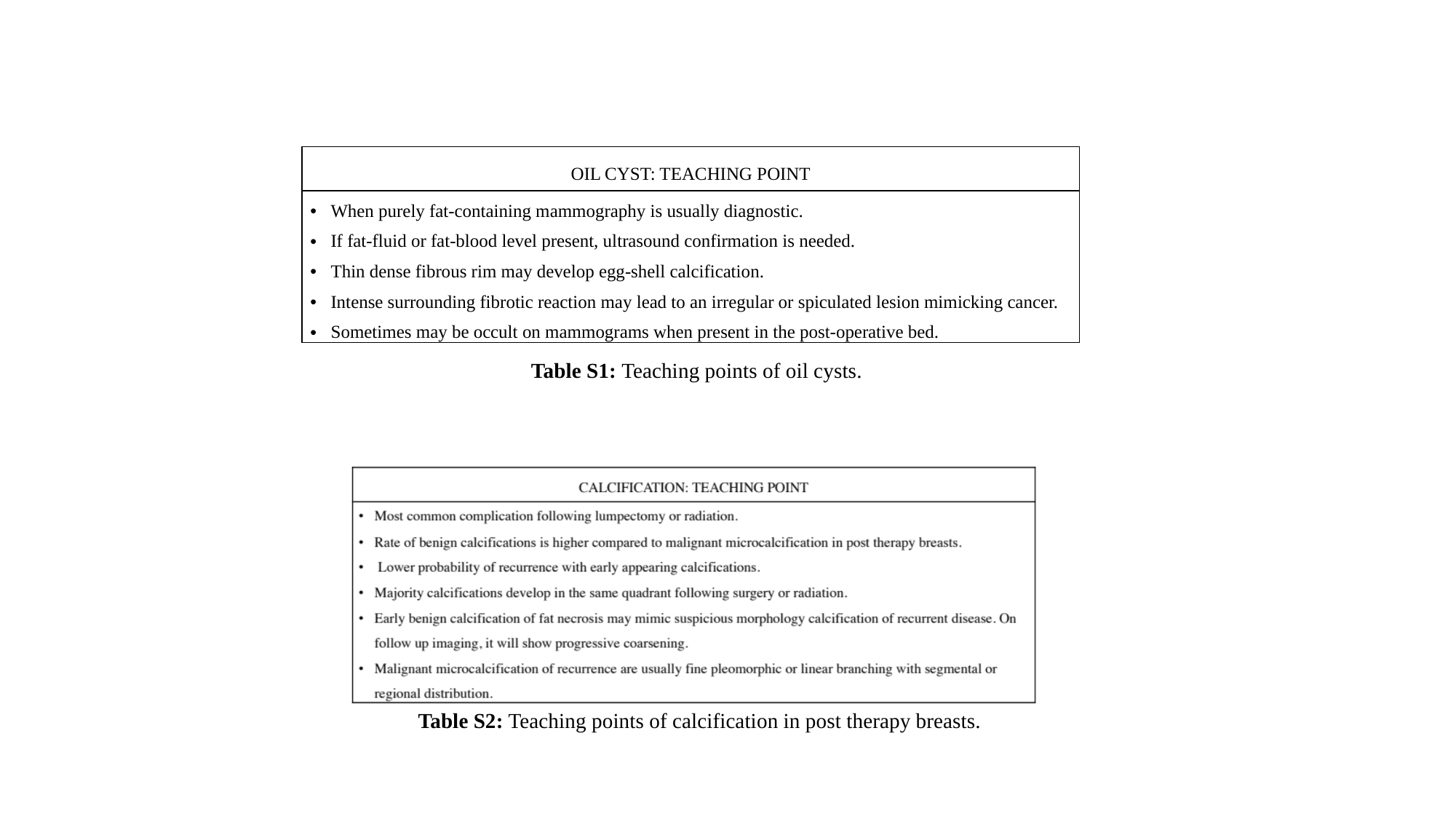

| OIL CYST: TEACHING POINT |
| --- |
| When purely fat-containing mammography is usually diagnostic. If fat-fluid or fat-blood level present, ultrasound confirmation is needed. Thin dense fibrous rim may develop egg-shell calcification. Intense surrounding fibrotic reaction may lead to an irregular or spiculated lesion mimicking cancer. Sometimes may be occult on mammograms when present in the post-operative bed. |
Table S1: Teaching points of oil cysts.
Table S2: Teaching points of calcification in post therapy breasts.

## Slide 3
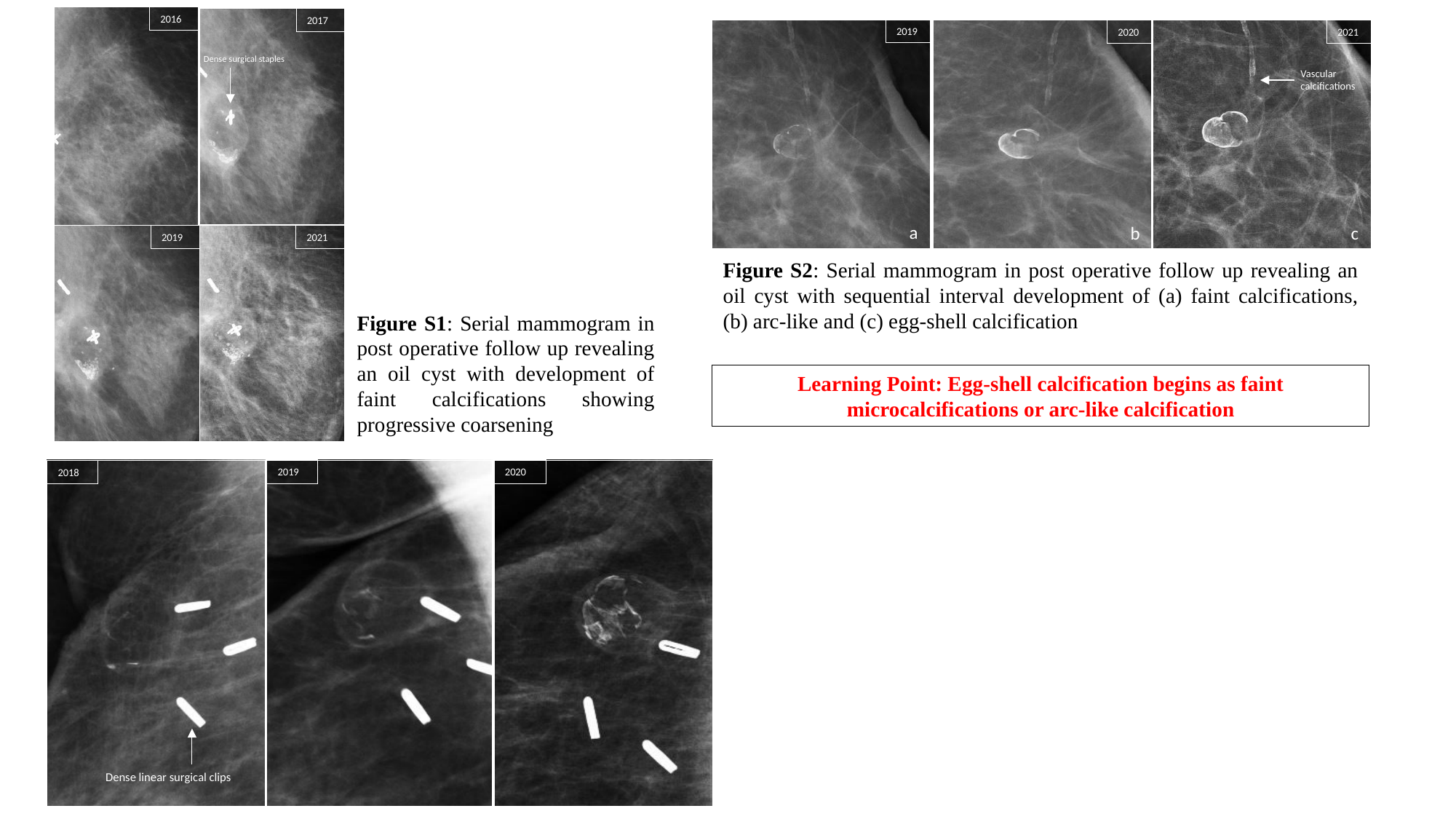

2016
2017
2019
2020
2021
Dense surgical staples
Vascular
calcifications
a
b
c
2019
2021
Figure S2: Serial mammogram in post operative follow up revealing an oil cyst with sequential interval development of (a) faint calcifications, (b) arc-like and (c) egg-shell calcification
Figure S1: Serial mammogram in post operative follow up revealing an oil cyst with development of faint calcifications showing progressive coarsening
Learning Point: Egg-shell calcification begins as faint microcalcifications or arc-like calcification
2020
2020
2019
2018
Dense linear surgical clips

## Slide 4
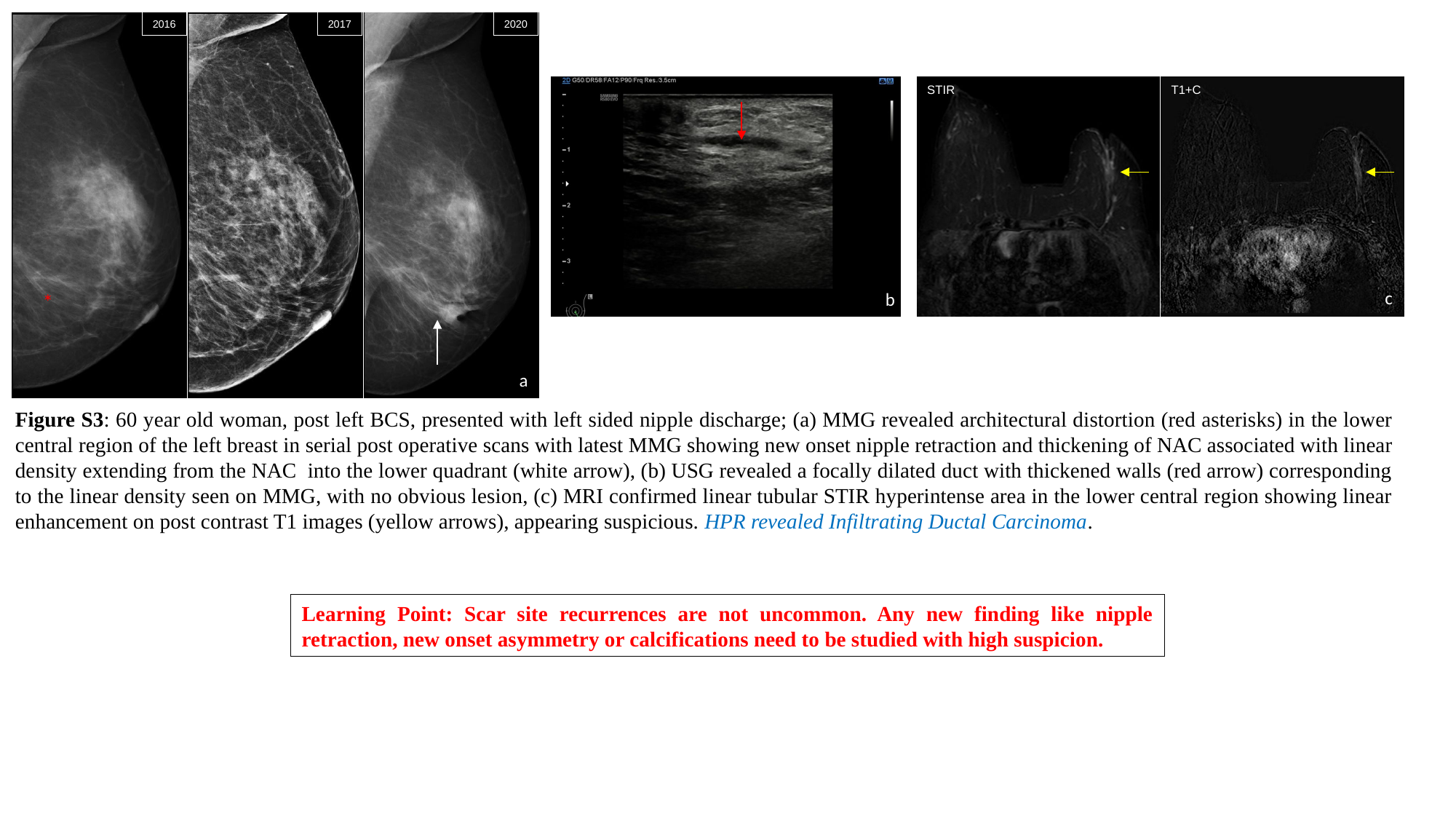

2016
2017
2020
STIR
T1+C
c
b
*
a
Figure S3: 60 year old woman, post left BCS, presented with left sided nipple discharge; (a) MMG revealed architectural distortion (red asterisks) in the lower central region of the left breast in serial post operative scans with latest MMG showing new onset nipple retraction and thickening of NAC associated with linear density extending from the NAC into the lower quadrant (white arrow), (b) USG revealed a focally dilated duct with thickened walls (red arrow) corresponding to the linear density seen on MMG, with no obvious lesion, (c) MRI confirmed linear tubular STIR hyperintense area in the lower central region showing linear enhancement on post contrast T1 images (yellow arrows), appearing suspicious. HPR revealed Infiltrating Ductal Carcinoma.
Learning Point: Scar site recurrences are not uncommon. Any new finding like nipple retraction, new onset asymmetry or calcifications need to be studied with high suspicion.

## Slide 5
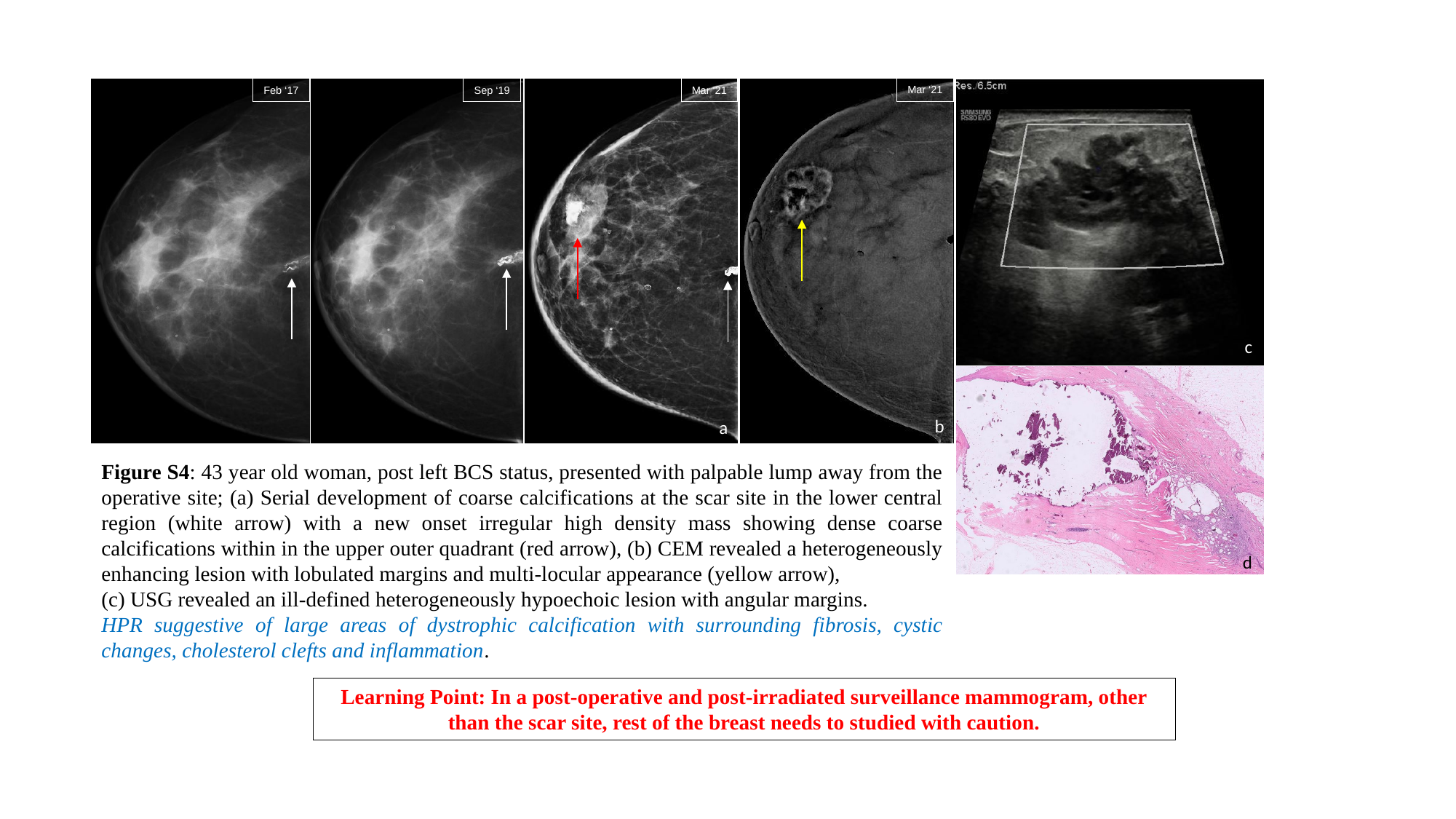

Mar ‘21
Feb ‘17
Sep ‘19
Mar ‘21
c
b
a
Figure S4: 43 year old woman, post left BCS status, presented with palpable lump away from the operative site; (a) Serial development of coarse calcifications at the scar site in the lower central region (white arrow) with a new onset irregular high density mass showing dense coarse calcifications within in the upper outer quadrant (red arrow), (b) CEM revealed a heterogeneously enhancing lesion with lobulated margins and multi-locular appearance (yellow arrow),
(c) USG revealed an ill-defined heterogeneously hypoechoic lesion with angular margins.
HPR suggestive of large areas of dystrophic calcification with surrounding fibrosis, cystic changes, cholesterol clefts and inflammation.
d
Learning Point: In a post-operative and post-irradiated surveillance mammogram, other than the scar site, rest of the breast needs to studied with caution.

## Slide 6
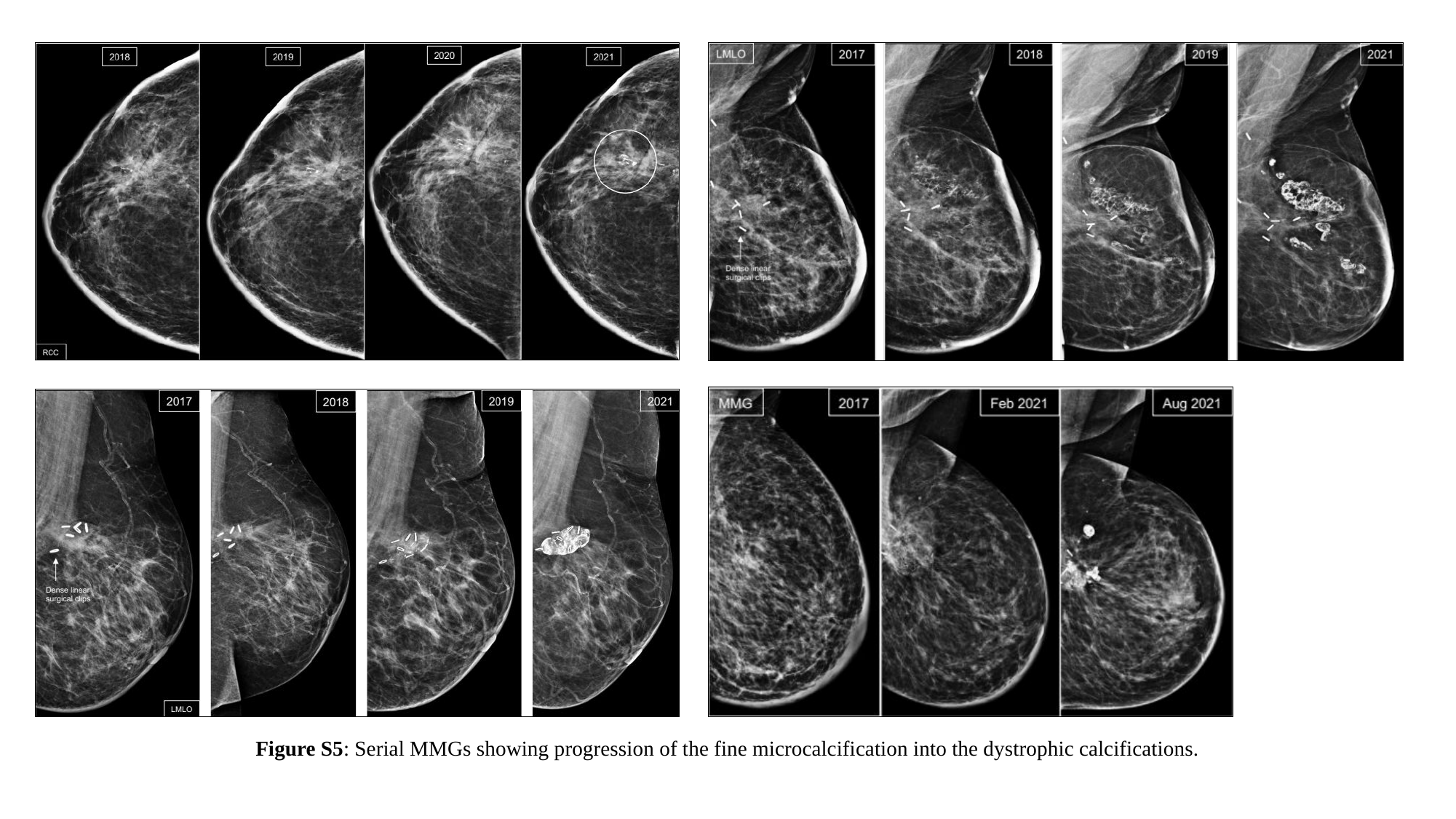

Figure S5: Serial MMGs showing progression of the fine microcalcification into the dystrophic calcifications.

## Slide 7
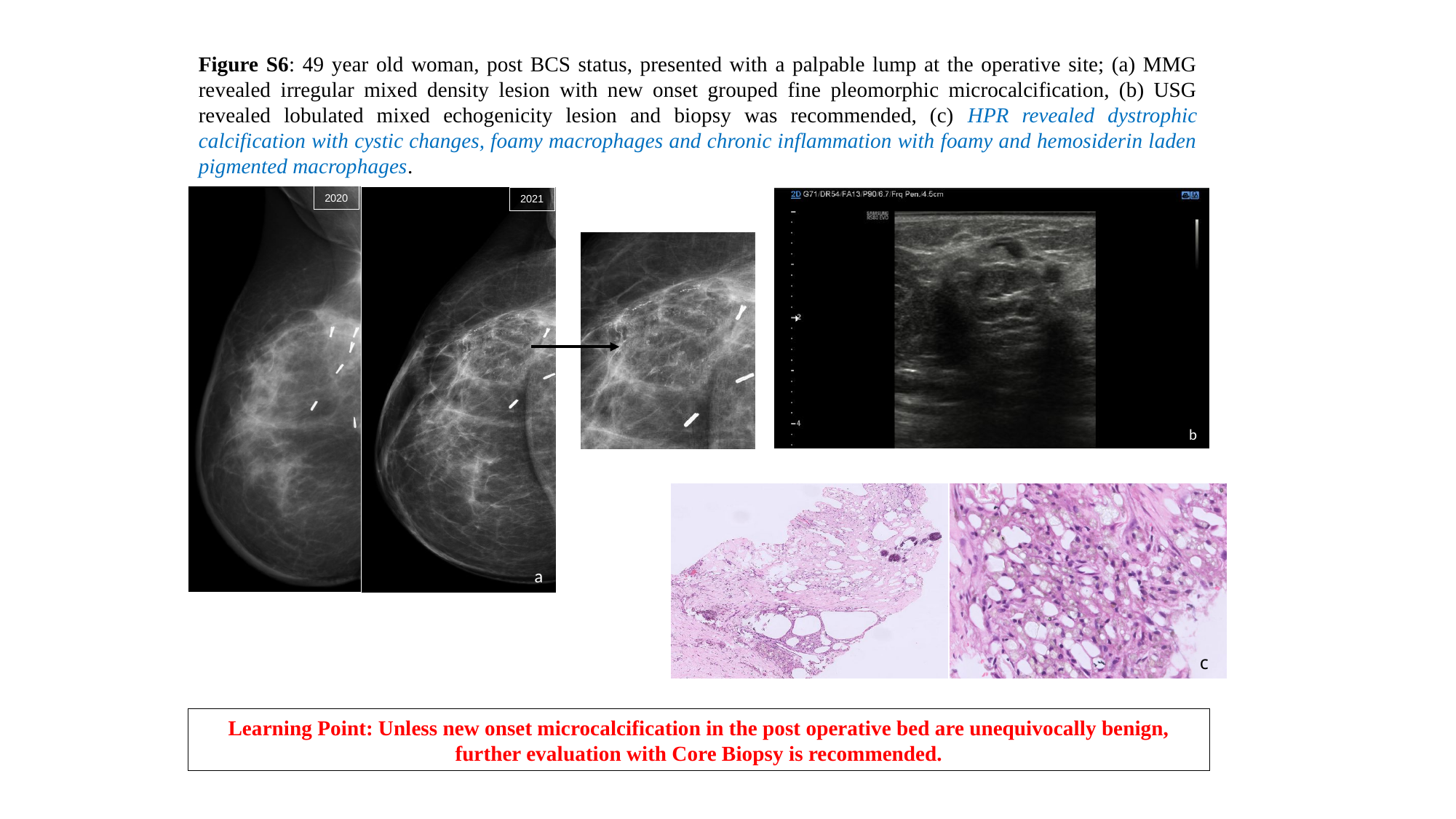

Figure S6: 49 year old woman, post BCS status, presented with a palpable lump at the operative site; (a) MMG revealed irregular mixed density lesion with new onset grouped fine pleomorphic microcalcification, (b) USG revealed lobulated mixed echogenicity lesion and biopsy was recommended, (c) HPR revealed dystrophic calcification with cystic changes, foamy macrophages and chronic inflammation with foamy and hemosiderin laden pigmented macrophages.
2020
2021
b
a
c
Learning Point: Unless new onset microcalcification in the post operative bed are unequivocally benign, further evaluation with Core Biopsy is recommended.

## Slide 8
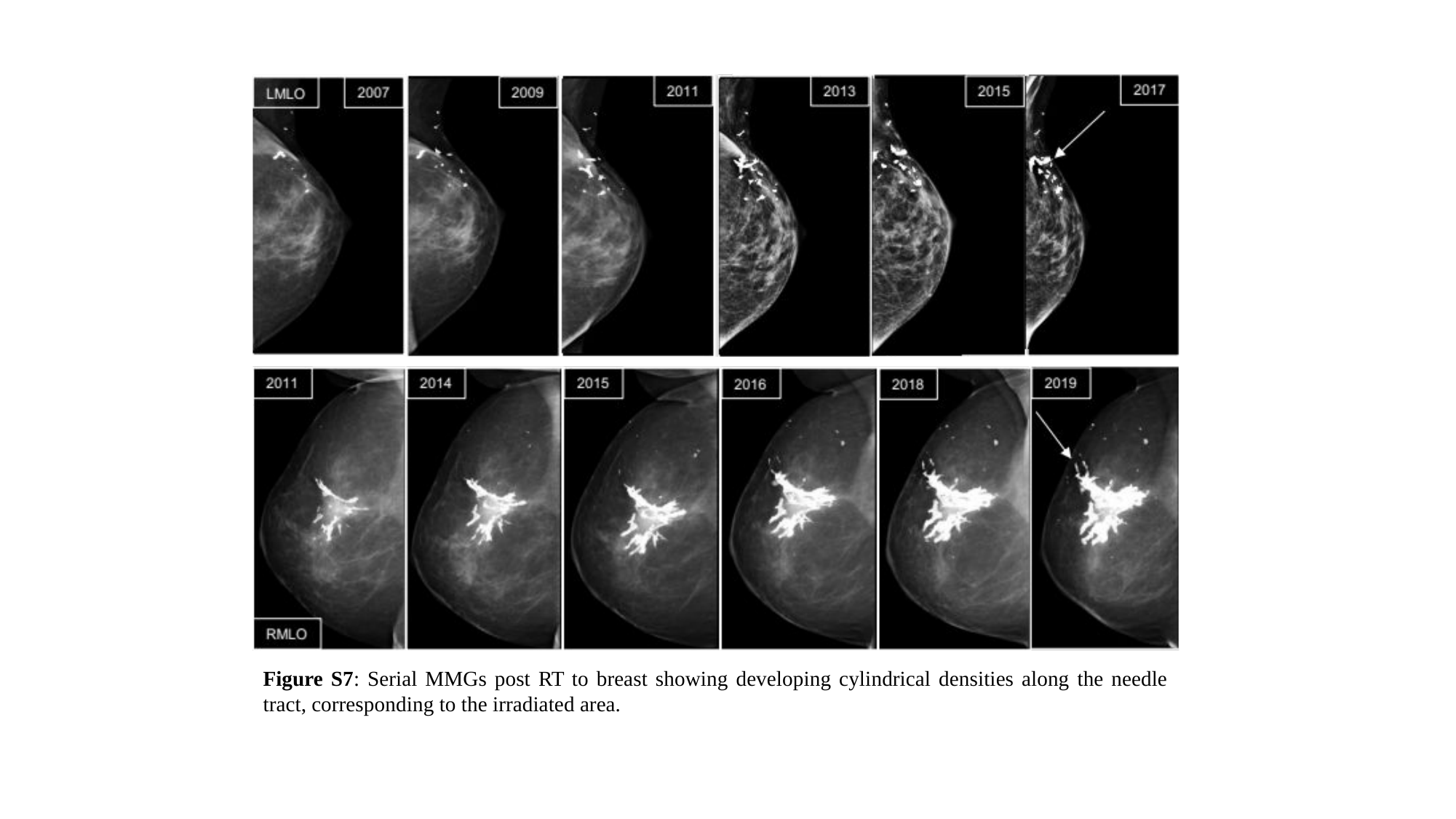

Figure S7: Serial MMGs post RT to breast showing developing cylindrical densities along the needle tract, corresponding to the irradiated area.

## Slide 9
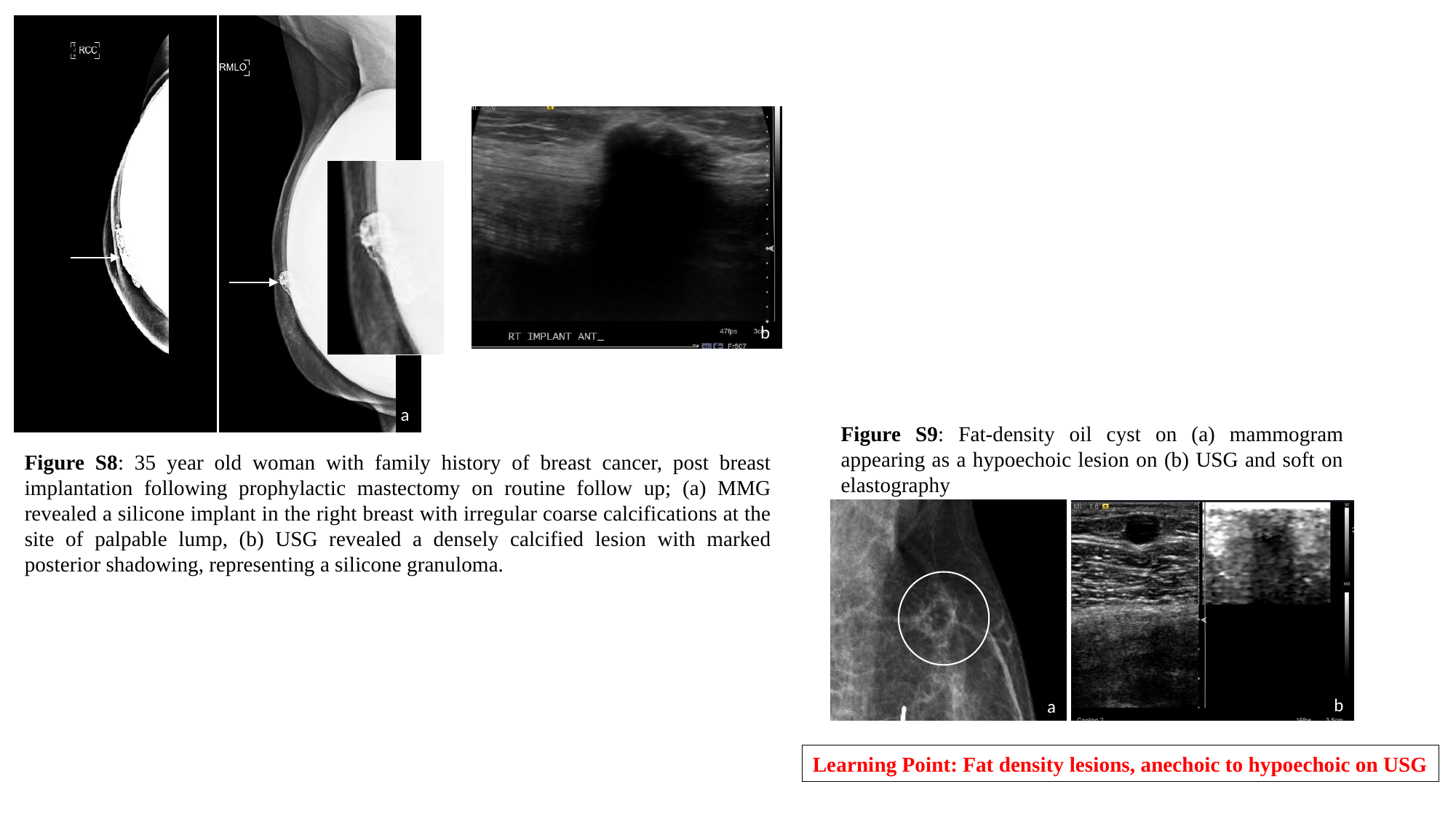

b
a
Figure S9: Fat-density oil cyst on (a) mammogram appearing as a hypoechoic lesion on (b) USG and soft on elastography
Figure S8: 35 year old woman with family history of breast cancer, post breast implantation following prophylactic mastectomy on routine follow up; (a) MMG revealed a silicone implant in the right breast with irregular coarse calcifications at the site of palpable lump, (b) USG revealed a densely calcified lesion with marked posterior shadowing, representing a silicone granuloma.
b
a
Learning Point: Fat density lesions, anechoic to hypoechoic on USG

## Slide 10
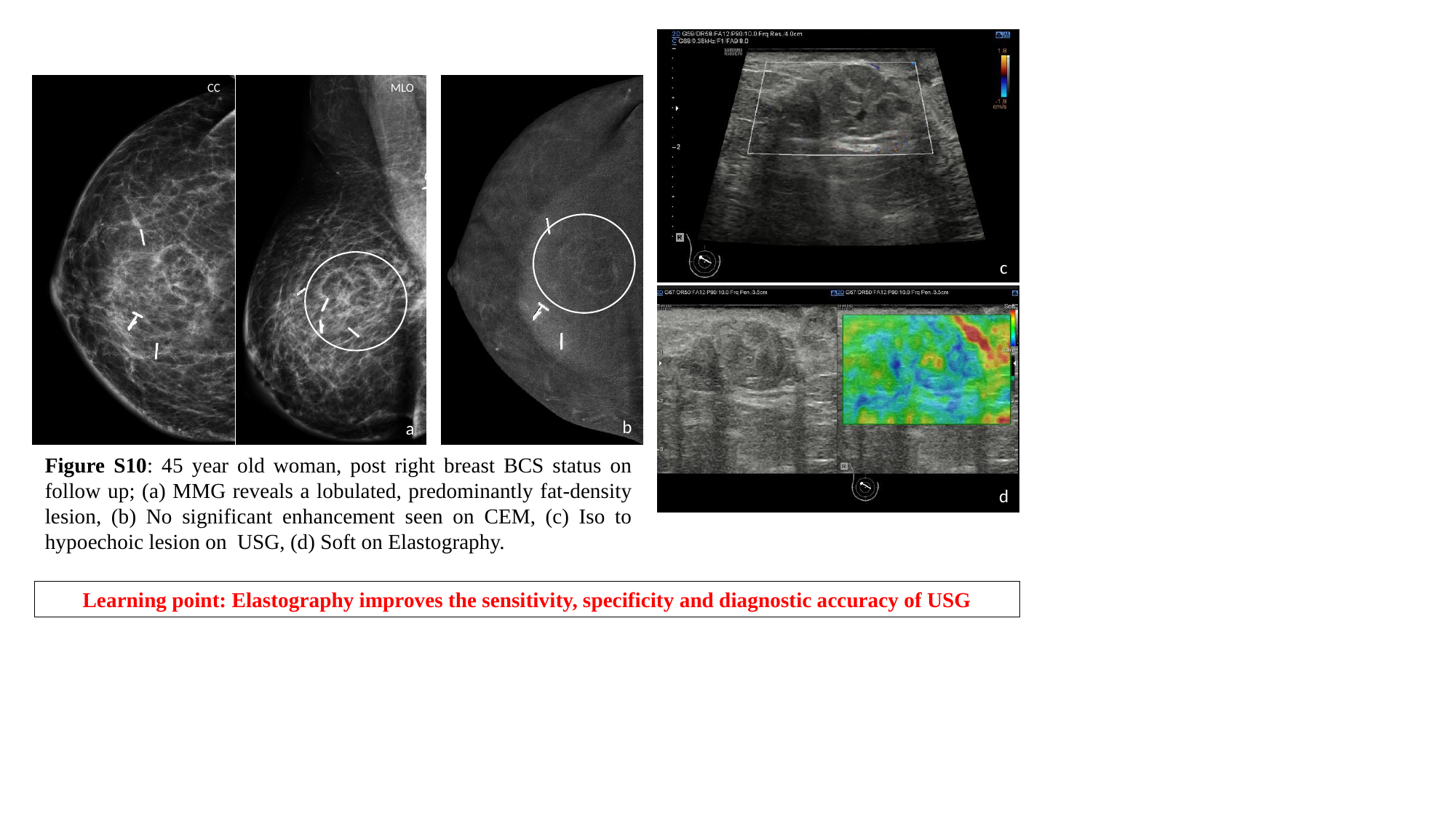

CC
MLO
c
b
a
Figure S10: 45 year old woman, post right breast BCS status on follow up; (a) MMG reveals a lobulated, predominantly fat-density lesion, (b) No significant enhancement seen on CEM, (c) Iso to hypoechoic lesion on USG, (d) Soft on Elastography.
d
Learning point: Elastography improves the sensitivity, specificity and diagnostic accuracy of USG

## Slide 11
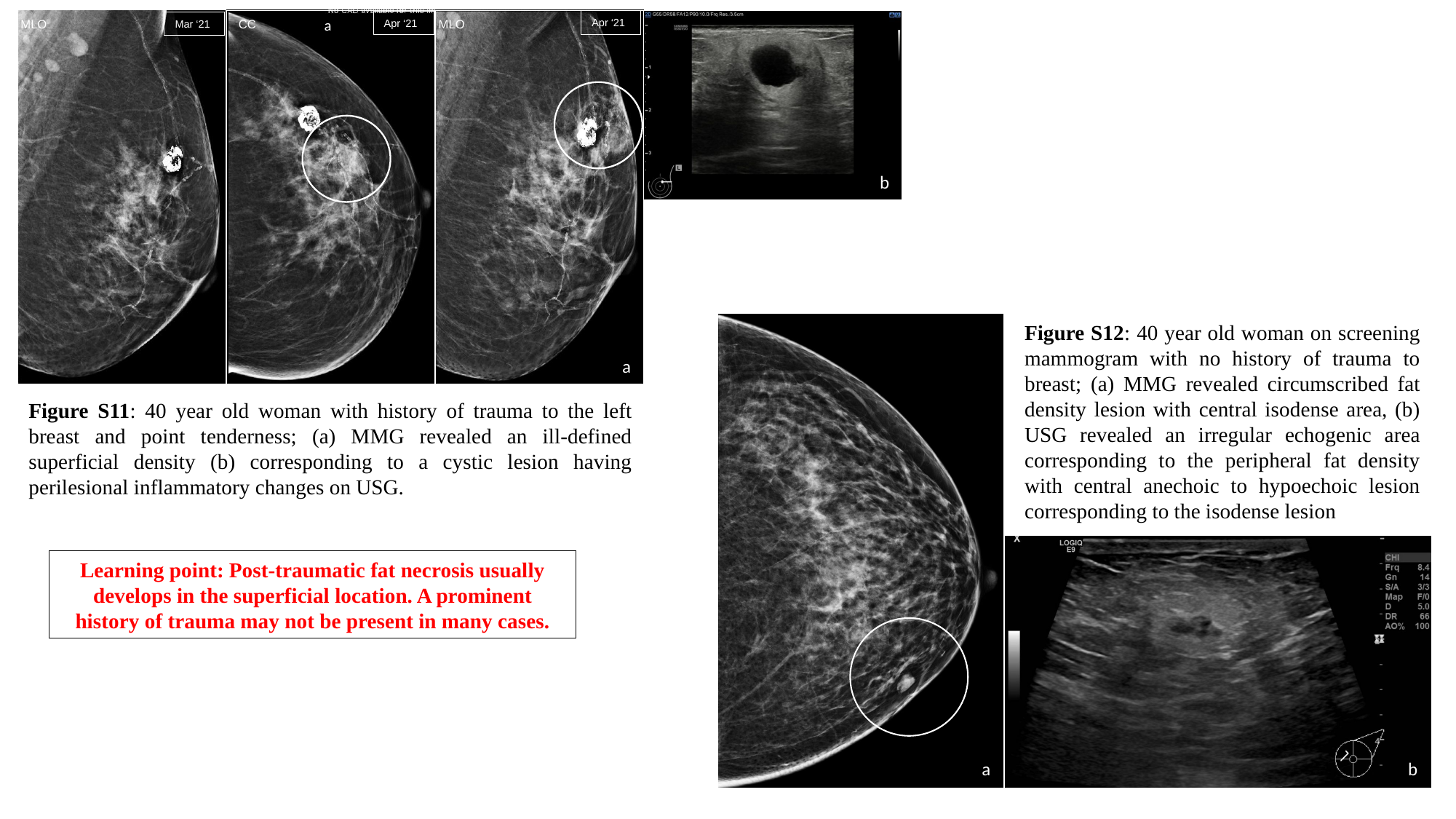

a
Apr ‘21
CC
MLO
Apr ‘21
MLO
Mar ‘21
b
Figure S12: 40 year old woman on screening mammogram with no history of trauma to breast; (a) MMG revealed circumscribed fat density lesion with central isodense area, (b) USG revealed an irregular echogenic area corresponding to the peripheral fat density with central anechoic to hypoechoic lesion corresponding to the isodense lesion
a
Figure S11: 40 year old woman with history of trauma to the left breast and point tenderness; (a) MMG revealed an ill-defined superficial density (b) corresponding to a cystic lesion having perilesional inflammatory changes on USG.
Learning point: Post-traumatic fat necrosis usually develops in the superficial location. A prominent history of trauma may not be present in many cases.
a
b

## Slide 12
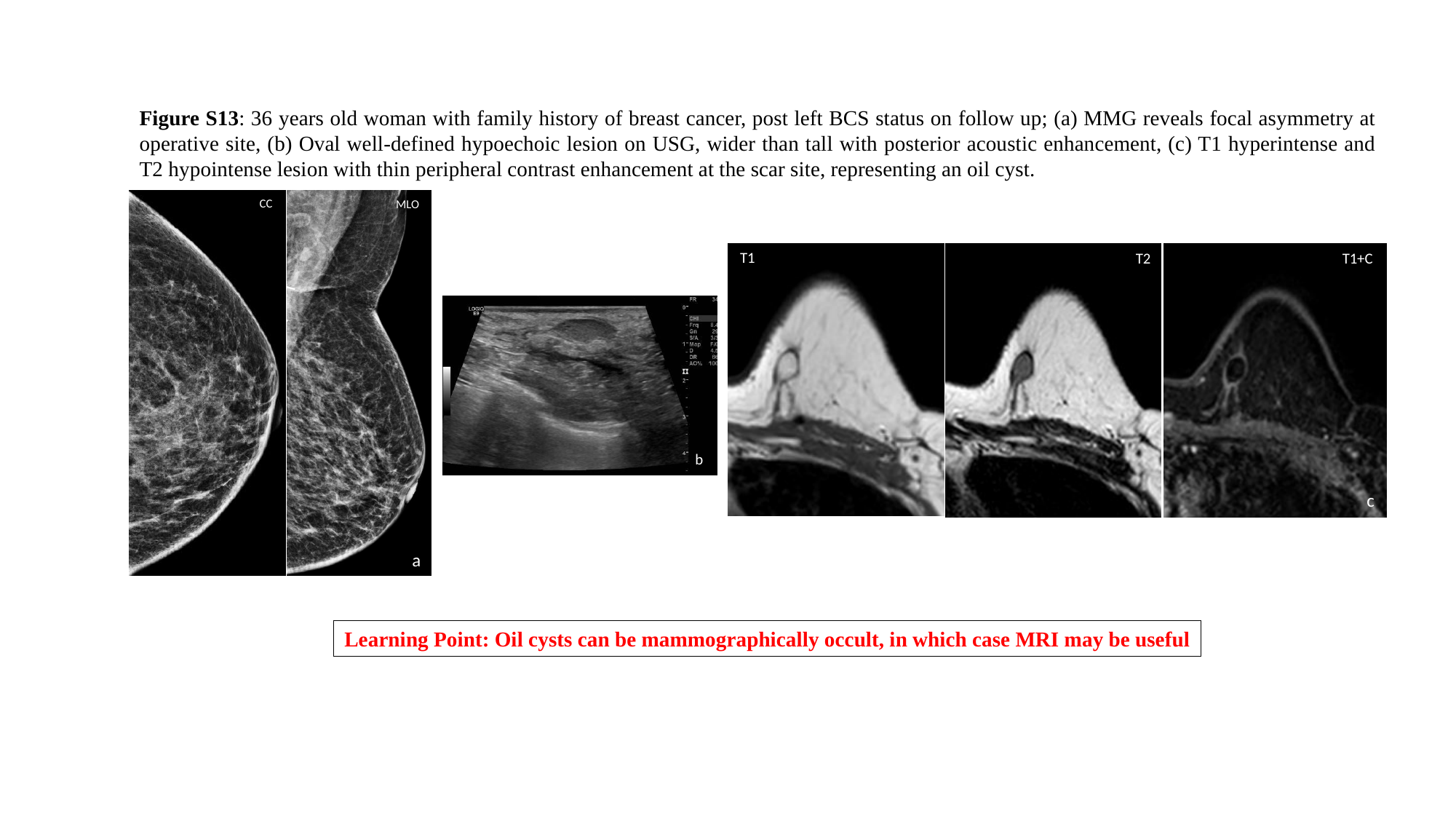

Figure S13: 36 years old woman with family history of breast cancer, post left BCS status on follow up; (a) MMG reveals focal asymmetry at operative site, (b) Oval well-defined hypoechoic lesion on USG, wider than tall with posterior acoustic enhancement, (c) T1 hyperintense and T2 hypointense lesion with thin peripheral contrast enhancement at the scar site, representing an oil cyst.
CC
MLO
T1
T2
T1+C
b
c
a
Learning Point: Oil cysts can be mammographically occult, in which case MRI may be useful

## Slide 13
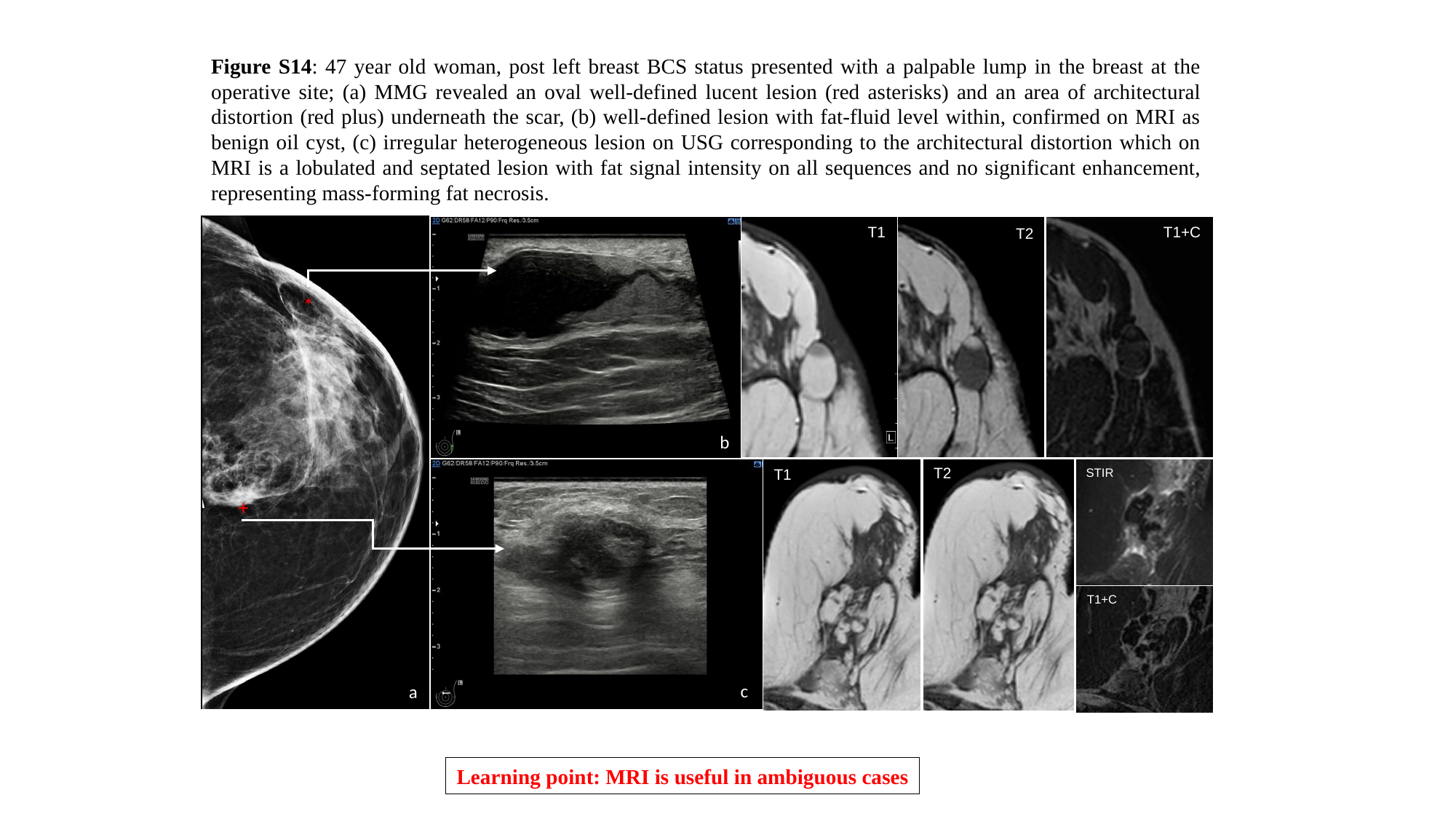

Figure S14: 47 year old woman, post left breast BCS status presented with a palpable lump in the breast at the operative site; (a) MMG revealed an oval well-defined lucent lesion (red asterisks) and an area of architectural distortion (red plus) underneath the scar, (b) well-defined lesion with fat-fluid level within, confirmed on MRI as benign oil cyst, (c) irregular heterogeneous lesion on USG corresponding to the architectural distortion which on MRI is a lobulated and septated lesion with fat signal intensity on all sequences and no significant enhancement, representing mass-forming fat necrosis.
T1
T1+C
T2
*
b
T2
T1
STIR
+
T1+C
c
a
Learning point: MRI is useful in ambiguous cases

## Slide 14
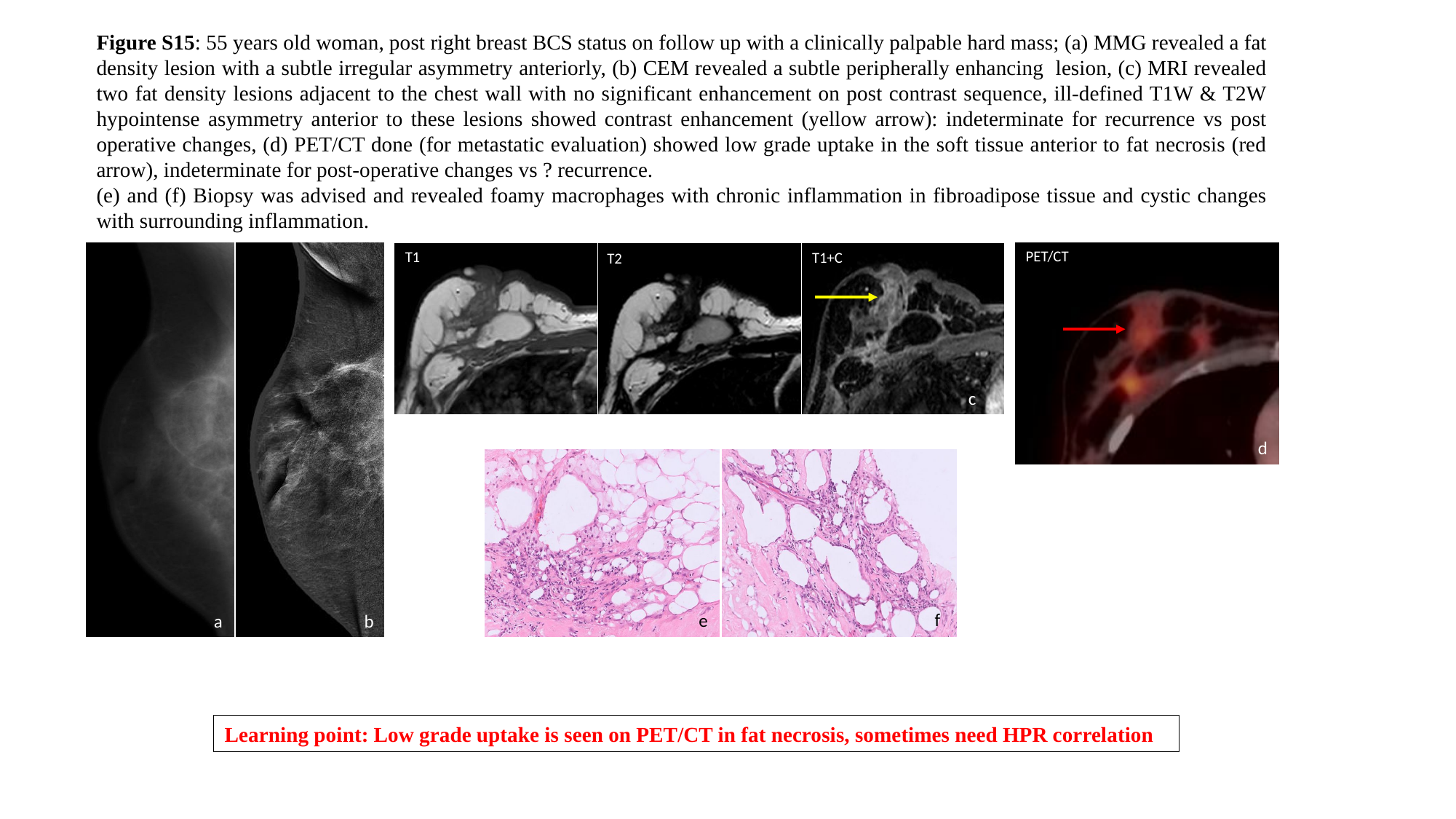

Figure S15: 55 years old woman, post right breast BCS status on follow up with a clinically palpable hard mass; (a) MMG revealed a fat density lesion with a subtle irregular asymmetry anteriorly, (b) CEM revealed a subtle peripherally enhancing lesion, (c) MRI revealed two fat density lesions adjacent to the chest wall with no significant enhancement on post contrast sequence, ill-defined T1W & T2W hypointense asymmetry anterior to these lesions showed contrast enhancement (yellow arrow): indeterminate for recurrence vs post operative changes, (d) PET/CT done (for metastatic evaluation) showed low grade uptake in the soft tissue anterior to fat necrosis (red arrow), indeterminate for post-operative changes vs ? recurrence.
(e) and (f) Biopsy was advised and revealed foamy macrophages with chronic inflammation in fibroadipose tissue and cystic changes with surrounding inflammation.
PET/CT
T1
T1+C
T2
c
d
f
e
a
b
Learning point: Low grade uptake is seen on PET/CT in fat necrosis, sometimes need HPR correlation
